# Meta‐Analysis of Mean Differences and Diagnostic Accuracy of Ptau‐217 in Amyloid‐Positive and Amyloid‐Negative Cognitively Unimpaired Older Adults

**DOI:** 10.1002/alz70856_096180

**Published:** 2025-12-24

**Authors:** Michael H. Malek‐Ahmadi, Savar Sharma, Fawaz Stipho, Nicholas J. Ashton

**Affiliations:** ^1^ Banner Alzheimer's Institute, Phoenix, AZ, USA; ^2^ Arizona Alzheimer's Consortium, Phoenix, AZ, USA; ^3^ University of Arizona College of Medicine‐Phoenix, Phoenix, AZ, USA; ^4^ Mountain Ridge High School, Glendale, AZ, USA; ^5^ University of Arizona College of Medicine‐Tucson, Tucson, AZ, USA; ^6^ Banner Sun Health Research Institute, Sun City, AZ, USA

## Abstract

**Background:**

Plasma markers of phosphorylated tau‐217 (ptau‐217) correlate well with in‐vivo AD pathology, however it is unclear how well this assay can differentiate cognitively unimpaired (CU) older adults who do and do not have AD pathology. The aim of this study was to evaluate the mean differences and diagnostic accuracy of ptau‐217 for published studies that compared amyloid‐positive and amyloid‐negative CU older adults.

**Method:**

Searches were carried out in PubMed, Embase, and Ebsco databases from inception to August 1, 2024. Observational studies or randomized clinical trials with baseline data on CU individuals who were classified as either amyloid‐positive or amyloid negative and reported numeric data for ptau‐217 levels. Two authors independently carried out literature searches to identify studies with CU older adults classified as either amyloid‐positive or amyloid‐negative where ptau‐217 was utilized. Hedge's g was used to characterize mean differences for ptau‐217. Pooled area under the curve (AUC) value was used to summarize the diagnostic accuracy of ptau‐217 in identifying amyloid‐positive individuals.

**Result:**

A total of 53 studies were screened of which 14 with 5,763 participants (2,313 amyloid‐positive, 3,450 amyloid‐negative) were included in the analysis of mean differences. Nine studies with 3,626 participants (1,697 amyloid‐positive, 1,929 amyloid‐negative) were included in the analysis of AUC values. The effect size for ptau‐217 mean differences was 1.45 (95% CI: 1.28, 1.61), indicating a large effect with high heterogeneity (I^2^ = 87%, 95% CI: 81%, 92%). The AUC meta‐analysis yielded an AUC 0.84 (95% CI: 0.80, 0.88) indicating that ptau‐217 has excellent diagnostic accuracy for identifying CU amyloid‐positive individuals. Subgroup analyses for assay platform, amyloid‐positive method, and study setting yielded similar results to the main findings. Thirteen of the studies used either the Meso Scale Development (MSD) or Single Molecular Array (Simoa) platform for ptau‐217 while one study used a liquid chromatography‐mass spectrometry platform.

**Conclusion:**

Plasma assays for ptau‐217 can accurately identify individuals in the preclinical phase of AD and the large effect size for mean differences between amyloid‐positive and amyloid‐negative individuals provides further support for the use of ptau‐217 as a diagnostic tool in identifying preclinical AD.

